# Nitric Oxide Donors and Selective Carbonic Anhydrase Inhibitors: A Dual Pharmacological Approach for the Treatment of Glaucoma, Cancer and Osteoporosis

**DOI:** 10.3390/molecules20045667

**Published:** 2015-03-31

**Authors:** Simone Carradori, Adriano Mollica, Celeste De Monte, Arianna Granese, Claudiu T. Supuran

**Affiliations:** 1 Department of Pharmacy, “G. D’Annunzio” University of Chieti-Pescara, Via dei Vestini 31, 66100 Chieti, Italy; E-Mail: a.mollica@unich.it; 2 Department of Drug Chemistry and Technologies, Sapienza University of Rome, P.le A. Moro 5, 00185 Rome, Italy; E-Mails: celeste.demonte@uniroma1.it (C.D.M.); arianna.granese@uniroma1.it (A.G.); 3 Laboratorio di Chimica Bioinorganica, Università degli Studi di Firenze, Via della Lastruccia 3, 50019 Sesto Fiorentino (Firenze), Italy; E-Mail: claudiu.supuran@unifi.it

**Keywords:** NO donors, selective carbonic anhydrase inhibitors, glaucoma, cancer, osteoporosis, dual pharmacological activity

## Abstract

Due to the recognized biological role of nitric oxide (NO) donating derivatives and of selective inhibitors of specific human carbonic anhydrase isoforms (CA, EC 4.2.1.1), promising compounds having an aromatic/heterocyclic primary sulfonamide and functionalized with NO-releasing moieties have been designed. These bifunctional agents have been tested *in vitro* and *in vivo* to assess their dual pharmacological activity. According to the encouraging results they could be proposed for the treatment of angle-open glaucoma, cancer regression and osteoporosis, in which both NO and CA activities are involved.

## 1. Introduction

A large number of complex and multifaceted diseases strictly require multipotent molecules concurrently able to modulate two or more mechanisms of action according to the “one molecule, multiple targets” theory [1]. Cancer, neurodegenerative conditions and cardiovascular problems are the most important examples in this field, due to their multifactorial nature of their aetiology and progression and, for this reason, an emerging effort is being oriented towards a Multi-Target-Directed Ligands approach (MTDL). The design of hybrid molecules incorporating moieties and pendants able to interact at different biological levels is an effective goal in order to reduce difficulties related to multiple drugs administration, pharmacokinetic aspects and low patient compliance. Researchers haave focused on several targets and produced a plethora of promising derivatives endowed with dual effects.

**Figure 1:**
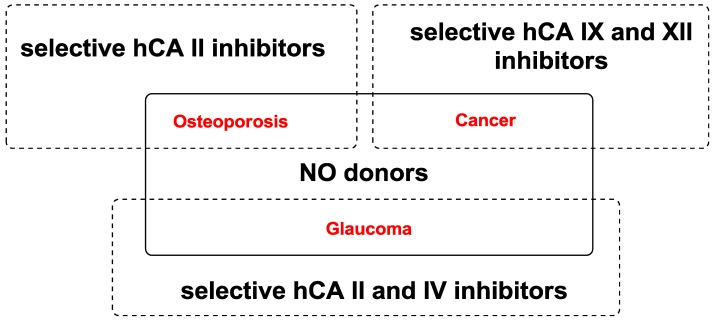
Diseases in which NO-donors and selective hCA inhibitors as dual pharmacological agents are involved.

In this review, we explored the state of the art of some molecules effective towards two pivotal targets: nitric oxide (NO) network and specific human carbonic anhydrase (hCA) isoforms (Figure 1).

The importance of NO is due to the fact that it plays a regulatory role in a wide array of biological effects, through or not the synthesis of cyclic guanosine monophosphate (cGMP) as a mediator [2]. The NO-regulated biochemical and physiological events are important in the regulation of cell functions because NO is one of the main intracellular messengers as well as a neurotransmitter not only for the cardiovascular system, but also for central nervous system (CNS), host’s immunity and response against tumor cells [3].

According to the reported properties, NO has a promising therapeutic potential and, currently, there is great interest in the design of NO-donating derivatives [4] for the transport and release of NO within the body. The NO-donors most commonly studied include organic nitrates (glyceryl trinitrate, mononitrate isosorbide) and NO-metal complexes (sodium nitroprusside), diazeniumdiolates, *S*-nitrosothiols and (benzo)furoxans [5]. Moreover, the design of hybrids containing NO-donating groups that act synergistically with the “native” drugs has attracted interest [6,7].

On the other side, human carbonic anhydrase isoforms are responsible for specific physiological functions in humans and their selective inhibitors could have innovative clinical applications as diuretic, antiglaucoma, antiobesity or antitumor drugs. Sulfonamide and sulfamate derivatives represent the main and most explored chemotypes of classical CA inhibitors [8,9,10,11,12], whereas other compounds (coumarins, polyamines, benzoic acids, natural compounds and phenols) were only recently evaluated for this pharmacological behavior [13,14,15]. Sulfonamide/sulfamate inhibitors usually bind in their deprotonated form to the catalytically Zn(II) ion in the enzymatic active site, participating also in many other interactions (hydrogen bond network, van der Waals contacts, p-stacking) with amino acid residues located both in the hydrophobic and hydrophilic halves of the active site as confirmed by X-ray crystallographic studies of enzyme-inhibitor complexes deposited for many adducts of several isozymes [16]. In consequence, the potent biological effects of specific sulfonamide-NO-donor compounds ultimately reported may be explained by the contribution of CA inhibition in addition to NO release.

## 2. Discussion

Most of the literature data reported the effect of lowering intraocular pressure (IOP) in patients with open-angle glaucoma [17], but emerging evidence also provided a beneficial role of these bifunctional agents in the treatment of specific cancers and osteoporosis as reported below.

### 2.1. Ocular Diseases

Open-angle glaucoma is a multifaceted disease associated with elevated IOP, damage of the optic nerve and loss of vision. IOP is based on the balance of aqueous humor production by the ciliary body and its drainage. The important role of NO in all compartments of the eye in maintaining basal resting tone of circulation has been recognized through cGMP-dependent pathways. Hypertensive glaucoma patients have a reduced NO/cGMP content in the aqueous humor. Despite these drugs are an underexplored class in the field of ocular therapeutics, glyceryl trinitrate decreases IOP and enhances cGMP levels *in situ*, likely for aqueous humor homeostasis in normal and pathological conditions [18,19].

Conversely, selective activation of hCA II, expressed in the ciliary bodies and elicited by β-adrenergic stimulation, improves aqueous formation and transport as well as its release into the posterior chamber, thus increasing IOP. For this reason, selective hCA II inhibitors are another class of drugs that has been used to reduce IOP [20,21]. Then, sulfonamide (compounds **1**–**16**) and atypical (compounds **17**–**28**) hCA inhibitors linked to NO-donors can be an interesting approach in cases of glaucoma, as reported in Table 1 [22,23,24]. hCA IV and overexpressed hCA XII isoforms are also found in the ciliary processes within the eyes of normal and glaucomatous patients [25,26] and showed to be innovative and less explored targets for this disease, whereas hCA I represents the main off-target isoform.

Accordingly, topical selective hCA II inhibitors such as dorzolamide (Trusopt^®^) or brinzolamide (Azopt^®^) are clinically used to regulate aqueous humor production and drainage and IOP in hypertensive glaucoma. On the contrary, hCA inhibitors such as acetazolamide (Diamox^®^), methazolamide, ethoxzolamide or dichlorophenamide have been used as systemic antiglaucoma drugs for more than 50 years, but suffered from side effects due to inhibition of the enzyme present in tissues other than the eye [27,28]: the possibility of a topical administration was also investigated, but with negative results. As a part of an ongoing research, some authors studied a new class of NO donating dorzolamide analogues (amides or carbamates) for topical administration that also presented inhibitory activity against hCA, such as compounds **1**–**5** reported in Table 1 [23]. These are examples of single molecules endowed with a dual mechanism of action (the enhancement of NO/cGMP signaling and the inhibition of specific CA isozymes). The NO-donating moieties comprehended an NO-donor linker (incorporating a nitrate ester) to the amine functionality of dorzolamide and were firstly evaluated for their stability to the hydrolysis by eye esterases in rabbit corneal homogenate. The dinitrate ester **5** was included to eventually measure the effect of increasing the theoretical deliverable amount of NO to eye tissues, whereas dorzolamide was the reference standard. Compounds **1**–**5** inhibited hCA II with *K*_i_ in the low nanomolar range. The alkyl nitrate linkers were more selective for hCA II than the benzyl nitrate spacer. Interestingly, compounds **1** and **3** were selective and equipotent to dorzolamide for hCA II. Conversely, hCA I inhibitory activity of these derivatives was lower (dorzolamide and isosorbide mononitrate were inactive). Compound **2** was also active against hCA IV in the low nanomolar range as dorzolamide. Crystallographic analysis of **5** in the CA active site showed some hydrogen bonds between secondary nitrate and carboxamide hydrogens of Asn62, between ester oxygen and carboxamide hydrogens and between primary nitrate oxygen with the imidazole moiety of His64.

**Table 1:** Dual agents endowed with NO donating properties and selective hCA isoform inhibitory activity for the treatment of open-angle glaucoma.

Compound	Structure	*K*_i_ (nM)		
		hCA I	hCA II	hCA IV
**Dorzolamide**	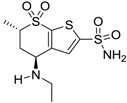	50,000	3.2	43
**Isosorbide mononitrate**	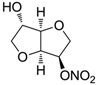	>100,000	>100,000	>100,000
**1**	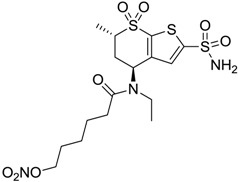	2950	14	4360
**2**	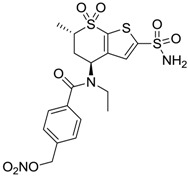	470	71	46
**3**	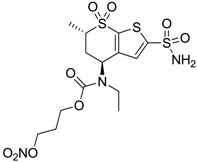	410	13	181
**4**	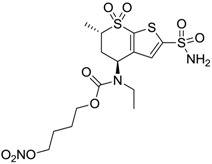	705	76	339
**5**	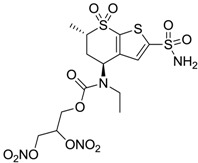	1520	63	3905
**6**	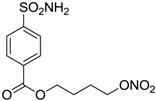	189	18	44
**7**	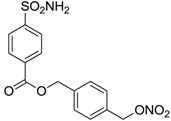	242	198	345
**8**	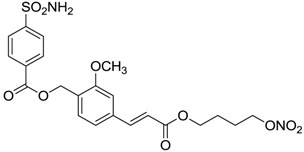	15	10	47
**9**	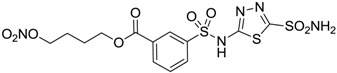	40	28	154
**10**	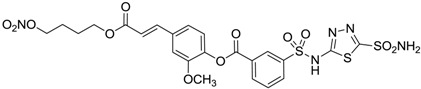	137	33	225
**11**	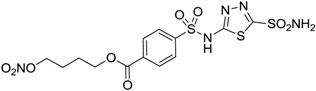	32	10	46
**12**	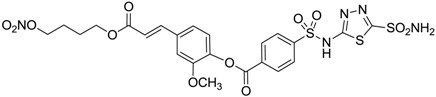	43	14	38
**13**	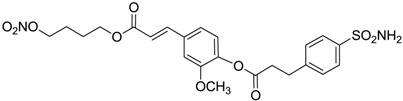	395	101	139
**14**	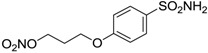	587	88	148
**15**	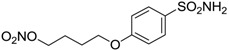	41	12	76
**16**	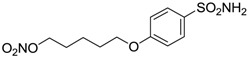	950	71	89
**17**	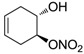	>100,000	>100,000	nd
**18**	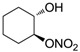	>100,000	>100,000	nd
**19**	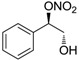	>100,000	>100,000	nd
**20**	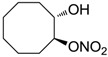	>100,000	>100,000	nd
**21**	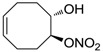	>100,000	>100,000	nd
**22**	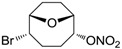	>100,000	>100,000	nd
**23**	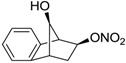	>100,000	>100,000	nd
**24**	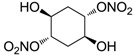	>100,000	>100,000	nd
**25**	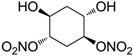	>100,000	>100,000	nd
**26**	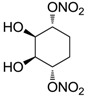	>100,000	>100,000	nd
**27**	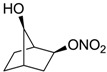	>100,000	>100,000	nd
**28**	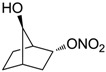	>100,000	>100,000	nd
**Dichlorophenamide**	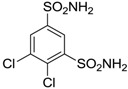	1200	38	15,000
**Acetazolamide**	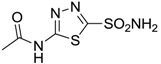	250	12	74

nd: not determined.

Another series of derivatives **6**–**16** was recently explored attaching “tails” to give better physico-chemical properties (water solubility, enhanced penetrability through the cornea) to five different sulfonamide scaffolds (4-carboxybenzenesulfonamide, 3- or 4-carboxybenzolamides, 4-(2-carboxyethyl)benzenesulfonamide and 4-hydroxybenzenesulfonamide) [22]. The hydroxyl or carboxyl moieties were functionalized by means of ester moieties (aliphatic C3–C5 moieties or the aromatic ones) releasing both NO and sulfonamides *in vivo*. Compounds **8**, **9**, **11**, **12** and **15** showed effective inhibition in the nanomolar range against the slow cytosolic isoform hCA I (an off-target enzyme for antiglaucoma agents), whereas the remaining compounds were less effective inhibitors. Other new sulfonamides acted as very good inhibitors of hCA II isozyme, with *K*_i_s in the range of 10–33 nM, in the same range as those of the systemically used antiglaucoma sulfonamides acetazolamide and dichlorophenamide or the topically acting dorzolamide. hCA IV was also effectively inhibited by some of them with respect to the clinically used drugs.

The last series of compounds **17**–**28** lack the sulfamoyl zinc-binding group and represent a class of mono- or polycyclic organic nitrate esters. Unfortunately, despite slight hCA II selectivity, their IC_50_ values ranged between 7.13 mM and 124 mM against hCA I and between 65.1 μM and 0.79 mM against hCA II (Table 1) [24]. hCA IV inhibitory activity for these compounds was not evaluated so far.

### 2.2. Cancer and Metastasis

The exact role of NO in the physiopathology of tumors has not achieved a complete consensus on the basis of its effects in numerous essential signaling pathways in tumor cells [29]. An emerging idea is that the capacity of NO to stimulate or inhibit cancer proliferation is related to its concentration, causing opposite effects. In detail, high expression or exogenous presence of NO could promote an antitumor effect on specific tumor cell lines by nitrosative stress involving the role of p53 leading to apoptosis [30]. A large number of studies also correlated the NO network with a high incidence of metastases in some cancers. Angiogenesis, proliferation and metastasis can normally be co-adjuvated by low levels of NO (<100 nM), whereas high concentrations of NO (>400–500 nM) may hamper tumor progression promoting cytotoxicity and cell apoptosis [31]. In this regard, NO could be considered a potent pharmacological tool in tumor-specific applications [32].

Conversely, the selective sulfonamide-based hCA IX inhibitors were shown to exert antiproliferative effects in tumor cell lines cultured in hypoxic conditions. This *in vivo* proof of concept confirmed the strong tumor retardation (in mice with xenografts of a renal clear cell carcinoma line, SK-RC-52) in animals treated for one month with these selective hCA IX inhibitors [16].

For the above mentioned reasons, some primary sulfonamide compounds have been preliminary investigated both as NO-donors and selective inhibitors of transmembrane hCA IX and XII isoforms that are almost overexpressed in hypoxic tumors as part of the network that cancer cells use to make the microenvironment favourable for solid tumor growth (Table 2).

**Table 2:** Dual agents endowed with NO donating properties and selective hCA isoform inhibitory activity for the treatment of cancer.

Compound	Structure	*K*_i_ (nM)	
		hCA IX	hCA XII
**Dorzolamide**	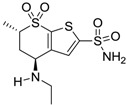	36	3.1
**6**	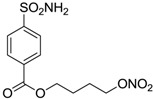	165	30
**7**	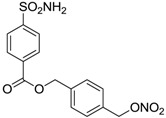	253	147
**8**	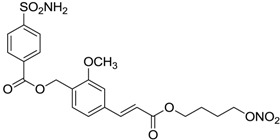	11	19
**9**	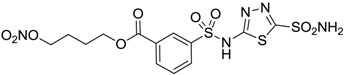	235	31
**10**	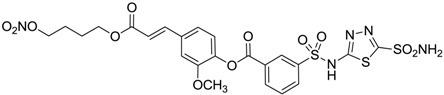	177	42
**11**	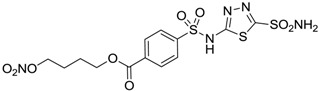	10	31
**12**	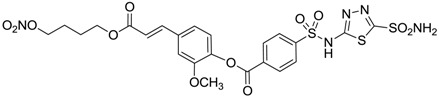	11	43
**13**	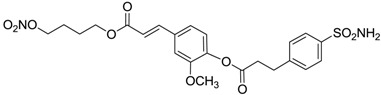	93	87
**14**	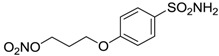	83	83
**15**	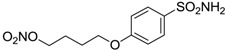	25	29
**16**	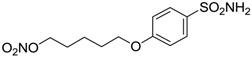	85	116
**Acetazolamide**	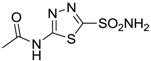	25	5.7

From the reported data it is possible to extrapolate that the tumor-associated hCA IX and XII isozymes were efficaciously inhibited by compounds **6**–**16** in the low nanomolar range (Table 2). Against hCA IX the best inhibitors were **8**, **11**, **12**, and **15**, which showed *K*_i_s in the range of 10–25 nM. They are characterized by an optimum distance between SO_2_NH_2_ and ONO_2_ groups on the basis of the chemical nature of the linker. Another group of derivatives (compounds **13**, **14** and **16**) were of medium inhibitory efficiency. Collectively, derivatives **6**–**16** were also effective as hCA XII inhibitors (*K*_i_s in the range of 19–147 nM) [22], contributing with new information about the structural requirements needed for the inhibition of this less known target. Unfortunately, from the comparison of the results reported in Table 1, it is evident that compounds **6**–**16** generally lack a strong inhibitory selectivity towards hCA IX and XII (a common issue with sulfonamide-type inhibitors) with respect to the other off-target isoforms. Moreover, for these compounds, no *in vivo* studies have been performed so far demonstrating this biological activity under hypoxic conditions.

### 2.3. Osteoporosis

While the effects of NO on osteoblasts are less characterized claiming the direct stimulation of osteoblast (growth, functioning and differentiation), regulation of bone formation and prevention of bone loss associated with the use of glucocorticoids [33], the role of selective hCA II inhibitors has been recognized recently [34]. Cellular and animal experiments led to observational studies and randomized trials that confirm the beneficial effects of organic nitrates on bone in men and women. Sulfonamide compounds with good hCA II isoform selectivity (Table 1), represent good lead compounds for the development of potential dual pharmacological drugs for the management of osteoporosis. Additional *in vivo* data are strongly required for these derivatives to support this dual approach.

## 3. Biological Evaluation *in vivo*

Compounds **1**, **3** and **6** were also characterized for NO release by the analysis of their cGMP-mediated vasorelaxant properties on methoxamine-precontracted rabbit aortic rings and their EC_50_ were better than those of isosorbide mononitrate and dorzolamide used as comparators at clinically relevant doses (Table 3). It may be observed that treatment with dorzolamide led to a maximal IOP decrease of 7.4 mmHg at 60 min. On the contrary, isosorbide mononitrate provided an IOP reduction of 6.8 mmHg, but the maximal effect was observed locally at 30 min post-administration.

**Table 3:** Intraocular pressure (IOP) lowering effects of dorzolamide, isosorbide mononitrate and selected compounds (**1**, **3** and **6**) in normotensive New Zealand white rabbits.

Compound *	IOP Lowering Effect				
	Basal IOP (mmHg)	After Carbomer (mmHg)	Post-Treatment IOP (mmHg) ^#^	ΔΔ_IOP_ (mmHg) **	*T*_max_ (min) ***
**1**	19.8 ± 0.7	-	15.9 ± 0.7	−4.7 ± 0.7	60
**3**	19.2 ± 0.5	-	16.5 ± 0.4	−3.7 ± 0.3	120
**6**	18.2 ± 0.7	32.4 ± 1.4	20.6 ± 1.8	−15.7 ± 1.8	60
**Vehicle**	17.9 ± 0.3	32.4 ± 1.3	31.8 ± 1.9	0 ± 09	-
**Isosorbide mononitrate**	16.8 ± 0.3	35.3 ± 1.2	28.4 ± 1.6	−6.8 ± 0.5	30
**Dorzolamide**	14.7 ± 0.9	35.4 ± 0.8	16.7 ± 0.8	−7.4 ± 1.9	60

* All drugs were administered at the final concentration of 2% in a volume of 50 μL. IOP was recorded before treatment (basal IOP) and at 30, 60, 90, 180 and 240 min thereafter, using an applanation tonometer (Tono-Pen XL-Medtronic, Solan). Values are reported as mean ± SEM of 8 rabbits per group. Carbomer 0.25% (THEA Pharmaceutical S.R.) 0.1 mL was introduced bilaterally into anterior chamber of preanesthetized rabbits (Zoletil 100, 0.10 mg/Kg b.w.) for inducing the ocular hypertension, 2–3 weeks before the treatment. ^#^ Post-treatment IOP is that reflecting the lowest measurement recorded over the observation period. ** ΔΔ_IOP_ reflects the maximal difference recorded in drug-treated *vs.* vehicle (Cremophor EL 5% and DMSO 0.4% in phosphate buffer pH 6.00 at room temperature). *** The time after administration when the maximal IOP lowering was achieved.

Both compounds **1** and **3**, which displayed limited solubility, were potent IOP-lowering agents as well as dorzolamide and better than the NO-donor isosorbide mononitrate in normotensive rabbits, providing the maximal response in the range of 30–120 min post-dosing. Compound **6** was characterized by good hCA II inhibition (and acceptable selectivity against off-target hCA I and IX isoforms) and higher water solubility for the topical administration, compared to other proposed sulfonamides (*i.e.*, **8** was a better *in vitro* hCA II inhibitor but with lower water solubility). The NO-donor sulfonamide **6** showed a very strong IOP lowering effect at 60 min post-administration, such as for the reference drug dorzolamide in this animal model of glaucoma. Its biological effect could be attributed both to selective and potent CA inhibition (as regards the dorzolamide IOP reduction) and the NO-mediated action (as regards the non-CA inhibitor NO-donor isosorbide mononitrate).

## 4. Conclusions

Multifactorial diseases must be treated with a combination of drugs acting at different biological levels. In order to reduce side-effects, low compliance and pharmacokinetic issues, the possibility of incorporating two putative mechanisms of action (usually due to distinct functional groups) in only one molecule is the goal of the recent medicinal chemistry efforts pursuing the Multi-Target-Directed Ligands approach (MTDL). In this short review, we have analyzed some bifunctional agents characterized by primary sulfonamide groups and nitrate esters to provide selective hCA inhibition and NO release, respectively. These interesting derivatives have been studied *in vitro* and *in vivo* for the treatment of specific pathologies (open-angle glaucoma, cancer and osteoporosis) showing that their effects could have a therapeutic role. As reported, additional biological data especially about cancer and osteoporosis treatment are required. Furthermore, it has been demonstrated that their strong efficacy as hCA inhibitors could arise not only from the sulfonamide moiety, coordinating to the Zn(II) ion from the enzymatic active site, but also from the same release of NO, which concurrently may bind itself to the enzyme or nitrosylate it, and may contribute to CA inhibition.
